# The influence of self-reported history of mild traumatic brain injury on cognitive performance

**DOI:** 10.1038/s41598-022-21067-w

**Published:** 2022-10-11

**Authors:** Amaya J. Fox, Hannah L. Filmer, Paul E. Dux

**Affiliations:** School of Psychology, The University of Queensland, McElwain Building, Campbell Road, St Lucia, QLD 4072 Australia

**Keywords:** Psychology, Human behaviour

## Abstract

The long-term cognitive consequences of mild traumatic brain injury (mTBI) are poorly understood. Studies investigating cognitive performance in the chronic stage of injury in both hospital-based and population-based samples have revealed inconsistent findings. Importantly, population-based mTBI samples remain under-studied in the literature. This study investigated cognitive performance among individuals with a history of self-reported mTBI using a battery of cognitively demanding behavioural tasks. Importantly, more than half of the mTBI participants had experienced multiple mild head injuries. Compared to control participants (*n* = 49), participants with a history of mTBI (*n* = 30) did not demonstrate deficits in working memory, multitasking ability, cognitive flexibility, visuospatial ability, response inhibition, information processing speed or social cognition. There was moderate evidence that the mTBI group performed better than control participants on the visual working memory measure. Overall, these findings suggest that even multiple instances of mTBI do not necessarily lead to long-term cognitive impairment at the group level. Thus, we provide important evidence of the impact of chronic mTBI across a number of cognitive processes in a population-based sample. Further studies are necessary to determine the impact that individual differences in injury-related variables have on cognitive performance in the chronic stage of injury.

## Introduction

Mild traumatic brain injury (mTBI), or concussion, is increasingly recognised as a significant public health problem^1^. Mild TBI occurs when biomechanical force transmitted to the head or body disrupts normal brain function (i.e., a short period of post-traumatic amnesia or confusion and/or disorientation)^2,3^. Worldwide, approximately 55.9 million people experience a mTBI each year^4^. Importantly, incidence rates for mTBI are typically derived from hospitalisation rates. Given that a large proportion of injuries go unreported or are diagnosed in the community (e.g., by general practitioners or medics), this incidence rate likely underestimates the actual occurrence of the injury^5^. Research investigating both the acute (< 3 months) and chronic (> 3 months) consequences of mTBI has increased substantially over the past two decades^6^. However, establishing the cognitive impacts of the injury, particularly in the long-term, remains a key area of investigation.

In the acute stage of mTBI, it is well-documented that individuals typically demonstrate impaired performance on measures assessing memory, attention, processing speed, and cognitive flexibility^7–10^. Conversely, research investigating between-group differences in cognitive performance in the chronic stage of mTBI has revealed conflicting findings. While some evidence suggests reduced cognitive performance in the above-mentioned domains persists in the months to years following injury^11,12^, other studies have observed no long-term deficits in mTBI populations^13,14^. Review papers exploring cognitive outcome in the chronic stage of mTBI have also revealed mixed findings. A recent scoping review determined that approximately half of individuals who experience a single mTBI show chronic cognitive impairment^15^; however, a later paper re-reviewed the same studies and established that a single incident of mTBI is not associated with a high rate of long-term cognitive impairment^16^. Notably, the latter conclusion aligns with the findings of other meta-analyses in this area^17,18^. Some variability in the findings across individual studies may be accounted for by the wide variety of tasks used to assess each cognitive domain, as some measures lack sufficient sensitivity to detect subtle long-term cognitive impairment following mTBI^19^.

Another important factor to acknowledge is the substantial variance in long-term outcome after mTBI^20^. For instance, cognitive outcome may be impacted by individual differences in injury factors (e.g., loss of consciousness^21^) and participant characteristics (e.g., cognitive reserve^22^). Measuring cognitive performance longitudinally from the point of injury is the best method for identifying the factors that predict poor outcome, as confounds typically present in cross-sectional studies (e.g., variable time since injury) are avoided. The few longitudinal studies in this area have begun to establish whether some individuals experience chronic cognitive impairment following mTBI; however, the extent and mechanisms underlying impairment remain poorly understood^23–26^. It is also important to note that many cross-sectional and longitudinal studies investigating cognitive performance in chronic mTBI have recruited samples from hospital settings. Given a significant number of individuals seek medical advice in the community or do not seek assistance at all after their injury, these findings may not be representative of the entire chronic mTBI spectrum^23^.

To address this, a few large-scale studies have investigated cognitive performance in individuals with a self-reported history of mTBI, which can capture both diagnosed and unreported injuries (i.e., population-based mTBI samples^27^). However, population-based mTBI samples are under-studied in comparison to clinical samples and the between-group comparisons that have been conducted have also revealed mixed findings. While a few studies have observed no long-term cognitive impact of self-reported mTBI^28,29^, other evidence suggests only mTBI participants with higher levels of post-concussion symptoms show impaired cognitive performance in the long-term^30–32^. More recently, studies with population-based samples have assessed cognitive performance via complex behavioural measures, rather than standard clinical measures. For example, university students with a self-reported history of mTBI from adolescence demonstrated decreased response inhibition compared to control participants on a Stroop task^33^. Similarly, Arciniega et al.^34^ reported persistent visual working memory deficits in undergraduate students in the chronic stage of mTBI, all of whom reported no residual symptoms of the injury. Across four change detection tasks, visual working memory impairment was revealed in different samples of roughly 32 individuals with a history of mTBI compared to control participants. Despite manipulating the presence of feedback, maintenance duration, and retrieval demands in a change detection paradigm, this impairment was consistently observed. This visual working memory deficit is yet to be replicated, and it remains unknown whether impairment extends to other cognitive domains, highlighting a promising avenue for the present research.

The current study investigated cognitive performance among individuals with a self-reported history of mTBI. Improving on existing chronic mTBI research, we used a series of complex behavioural tasks, rather than traditional clinical measures, to examine a broad range of cognitive domains. Specifically, to measure visual working memory, we adopted the change detection paradigm used by Arciniega et al.^34^, who found a negative impact of mTBI. To extend on this previous research and determine whether other cognitive domains are persistently impacted by mTBI, we also included tasks assessing verbal working memory, multitasking ability, cognitive flexibility, visuospatial ability, response inhibition, information processing speed and social cognition. We focused on measures of executive function (e.g., working memory, multitasking) and information processing speed as these domains are consistently investigated in the literature and generally show impairment in acute mTBI^17^. We also included visuospatial and social cognition tasks, as these domains are relatively under-investigated in the chronic mTBI population. Measures of post-concussion symptoms, depression, and anxiety were also administered, to determine whether these factors influenced cognitive function. In addition, our experimental design and proposed analyses were preregistered prior to data collection. To date, very few studies in this area have been preregistered despite the importance of this practice^35^. Therefore, it is possible that post hoc hypotheses and analyses may have impacted the reported findings in the current literature. Finally, this study used a Bayesian statistical approach. Bayesian analyses quantify relative support for both the null and alternative hypotheses given the observed data^36^, thus providing a more robust account of cognitive performance in the chronic stage of mTBI.

## Results

### Demographics and Questionnaire Measures

Demographic, emotional state, post-concussion symptom, and injury variables for each group are shown in Table 1. Importantly, there was weak to strong evidence against between-group differences for age, gender, and education. The two groups also did not differ in emotional state, with weak to moderate evidence against between-group differences for self-reported depression, anxiety, and stress. Regarding RPQ scores, there was weak evidence for a between-groups difference in post-concussion symptoms, with mTBI participants reporting only slightly more post-concussion symptoms than control participants. This aligns with past literature that reported similar rates of post-concussion symptoms in mTBI and healthy populations^37,38^ and supports the idea that post-concussion symptoms (e.g., headaches) are likely not specific to mTBI^39^.

**Table 1:** Demographic, emotional state, post-concussion symptom and mTBI information by group.

	mTBI group (*n* = 30)	Control group (*n* = 49)	BF_10_	*t*	*p*	*d*
Age	23.63 (6.97)	22.10 (3.91)	0.471	1.253	0.214	0.291
Gender (F/M)	17/13	33/16	0.428	0.914^a^	0.339	0.108^b^
Education (HS/B/M/O, %)	63.3/30.0/3.3/3.3	55.1/34.7/8.2/2.0	0.012	3.273^a^	0.513	0.204^b^
Depression (DASS-21)	3.53 (3.15)	3.86 (3.31)	0.260	− 0.430	0.669	− 0.100
Anxiety (DASS-21)	3.67 (2.93)	3.67 (2.95)	0.240	− 0.010	0.992	− 0.002
Stress (DASS-21)	5.70 (2.58)	5.02 (3.38)	0.352	0.945	0.348	0.219
RPQ	12.07 (9.83)	7.82 (7.97)	1.573	2.104	0.039	0.488
Time since injury (years)^c^	3.63 (3.36)	–	–	–	–	–
Number past mTBI	2 (1.22)	–	–	–	–	–
> 1 mTBI (%)	53.3	–	–	–	–	–
Loss of consciousness (%)^c^	36.7	–	–	–	–	–
Post-traumatic amnesia (%)^c^	50.0	–	–	–	–	–
Disorientation/confusion (%)^c^	93.3	–	–	–	–	–
Medical attention received (%)^c^	57.6	–	–	–	–	–
Cause, sports participation (%)^c^	40.0	–	–	–	–	–
Cause, motor vehicle accident (%)^c^	6.7	–	–	–	–	–
Cause, fall (%)^c^	20.0	–	–	–	–	–
Cause, head striking object (%)^c^	20.0	–	–	–	–	–
Cause, interpersonal violence (%)^c^	6.7	–	–	–	–	–
Cause, not disclosed (%)^c^	6.7	–	–	–	–	–

Standard deviations presented in parenthesis. Gender: *F* female, *M* male. Education: *HS* high school, *B* bachelor’s degree, *M* master’s degree, *O* other.

^a^Chi-square test performed.

^b^Cramer’s *V* reported.

^c^For most recent mTBI.

### Cognitive task performance

**Figure 1:**
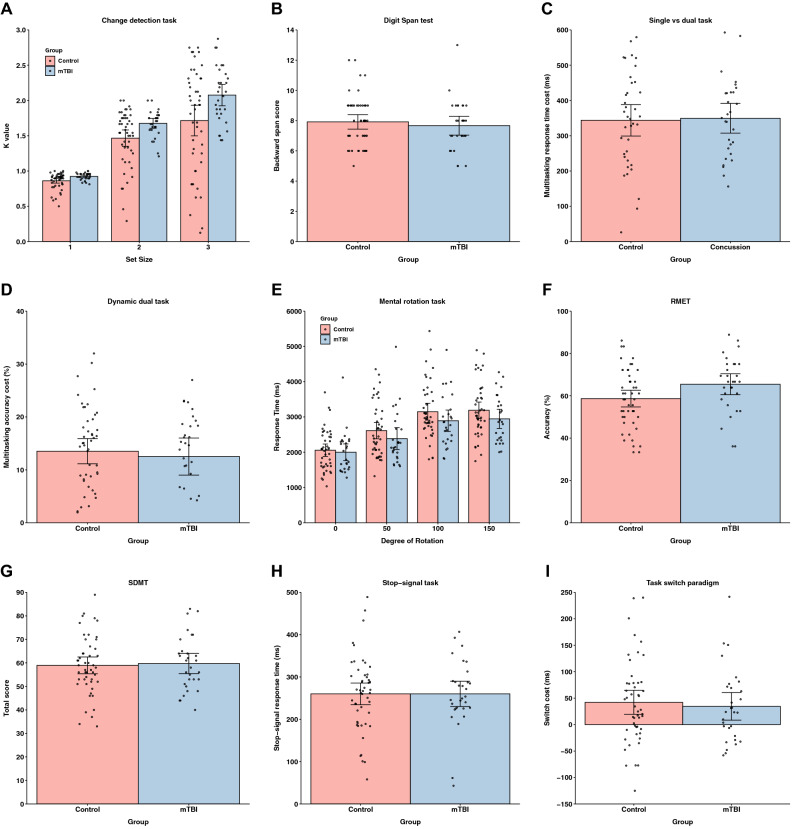
Cognitive task performance. Barplots depicting mean performance for (**A**) change detection task (K value), (**B**) digit span test (backward span score), (**C**) single vs dual task (multitasking reaction time cost; ms), (**D**) dynamic dual task (multitasking accuracy cost; %), (**E**) mental rotation task (response time; ms), (**F**) RMET (accuracy; %), (**G**) SDMT (total score), (**H**) stop-signal task (stop-signal response time; ms) and (**I**) task switch paradigm (switch cost; ms). Error bars indicate 95% confidence intervals. Individual data points are superimposed on each barplot.

Mean performance across all tasks is depicted in Fig. 1. Overall, the present findings provide evidence for no long-term cognitive performance deficits among individuals with a history of mTBI across all cognitive domains investigated.

Verbal working memory, information processing speed, response inhibition, multitasking ability, cognitive flexibility, and Theory of Mind appeared to not be impacted by mTBI history. A series of Bayesian independent samples t-tests were performed to investigate group differences on the key dependent measure for the Digit Span test, SDMT, stop-signal task, single vs dual task, dynamic dual task, task switch paradigm, and RMET (see Table 2). These analyses revealed moderate evidence against between-group differences in backward span scores on the Digit Span test, number of correct responses on the SDMT, stop-signal response time on the stop-signal task, multitasking response time cost on the single vs dual task, multitasking accuracy cost on the dynamic dual task, and switch cost on the task switch paradigm. Importantly, the typical multitasking cost (i.e., longer response times for dual compared to single task trials on the single vs dual task and lower accuracy for dual compared to single task trials on the dynamic dual task) and task switch effects (i.e., longer response times on task change compared to cue change trials on the task switch paradigm) were observed on these latter tasks (BF_incl_ > 4.615e+19, *F*s > 166.322, *p*s < 0.001). In contrast, there was weak evidence for a between-groups difference in accuracy on the RMET.

**Table 2:** Descriptive statistics, sample size, and independent samples *t*-tests for the digit span test, SDMT, stop-signal task, single vs dual task, dynamic dual task, task-switch paradigm, and RMET.

Task (key measure)	mTBI group		Control group		BF_10_	*t*	*p*	*d*
	*M* (SD)	*n*	*M* (SD)	*n*				
Digit span (backward span score)	7.67 (1.67)	30	7.92 (1.67)	49	0.288	0.651	0.517	0.151
SDMT (accuracy (total correct))	59.73 (11.60)	30	58.96 (12.41)	49	0.248	− 0.276	0.784	− 0.064
Stop-signal (stop-signal RT (ms))	260.10 (79.85)	30	260.10 (89.01)	49	0.240	0.001	0.999	3.389e−4
Single vs dual (multitasking RT cost (ms))	336.46 (89.34)	29	332.97 (76.94)	38	0.256	− 0.172	0.864	− 0.042
Dynamic dual (multitasking accuracy cost)	12.51 (9.39)	30	13.52 (8.27)	49	0.267	0.500	0.619	0.116
Task switch (switch cost (ms))	34.61 (69.83)	30	42.15 (79.23)	49	0.259	0.429	0.669	0.099
RMET (Accuracy (%))	65.46 (13.21)	30	58.67 (13.80)	49	1.729	− 2.156	0.034	− 0.500

*RT* response time.

Additionally, mTBI history does not appear to influence visuospatial ability (see Tables 3 and 4). Bayesian repeated measures ANOVAs were conducted on response time and error rate for the mental rotation task. The response time analysis revealed very strong evidence for a main effect of degree of rotation, reflecting the standard mental rotation effect. There was strong evidence for a main effect of trial type, indicating faster response times for same trials compared to different trials. Conversely, there was weak evidence against a main effect of group. There was strong evidence for a degree of rotation × trial type interaction, but moderate to strong evidence against a trial type × group interaction, a degree of rotation × group interaction and a degree of rotation × trial type × group interaction. The error rate analysis revealed similar findings, with strong to very strong evidence for main effects of degree of rotation and trial type, and weak evidence against a main effect of group. There was strong to decisive evidence for degree of rotation × trial type and trial type × group interactions, but very strong evidence against a degree of rotation × group interaction. There was only weak evidence for a degree of rotation × trial type × group interaction.

**Table 3:** Descriptive statistics and sample size for the key dependent measure for the mental rotation and change detection tasks.

Task (key measure)	mTBI group		Control group	
	*M* (SD)	*n*	*M* (SD)	*n*
Mental rotation (RT (ms) 0° rotation)	1998.96 (588.76)	26	2057.06 (577.29)	44
Mental rotation (RT (ms) 50° rotation)	2386.19 (768.80)	26	2612.04 (753.29)	44
Mental rotation (RT (ms) 100° rotation)	2892.79 (747.35)	26	3148.75 (787.21)	44
Mental rotation (RT (ms) 150° rotation)	2946.84 (676.23)	26	3189.21 (765.55)	44
Change detection (K set size 1)	0.92 (0.05)	30	0.86 (0.12)	48
Change detection (K set size 2)	1.68 (0.19)	30	1.46 (0.40)	48
Change detection (K set size 3)	2.08 (0.41)	30	1.68 (0.78)	48

*RT* response time.

**Table 4:** Repeated measures ANOVAs for the mental rotation and change detection tasks, *RT* response time.

Effects	BF_incl_	*F*	*p*	*η*_*p*_^*2*^
**Mental rotation (RT)**				
Degree of rotation	1.585e+63	153.318	< 0.001	0.693
Trial type	2.837e+8	34.104	< 0.001	0.334
Group	0.653	1.633	0.206	0.023
Degree of rotation × trial type	2.359e+23	56.178	< 0.001	0.452
Degree of rotation × group	0.082	1.119	0.343	0.016
Trial type × group	0.260	0.977	0.326	0.014
Degree of rotation × trial type × group	0.056	0.372	0.773	0.005
**Mental rotation (effect size)**				
Degree of rotation	4.939e+14	31.973	< 0.001	0.320
Trial type	7.030e+11	26.771	< 0.001	0.282
Group	0.797	3.598	0.062	0.050
Degree of rotation × trial type	12.976	5.386	0.001	0.073
Degree of rotation × group	0.018	0.041	0.989	6.006e–4
Trial type × group	13,998.875	14.238	< 0.001	0.173
Degree of rotation × trial type × group	1.523	3.478	0.017	0.049
**Change detection (K)**				
Set size	8.243e+37	186.504	< 0.001	0.710
Group	6.595	7.763	0.007	0.093
Set size × group	5.499	5.334	0.006	0.066

In line with our preregistered analyses, we also ran Bayesian repeated-measures ANOVAs on mental rotation response time and error rate for each trial type separately. All analyses revealed strong to very strong evidence for a main effect of degree of rotation (BF_incl_ > 3.449e + 6, *F*s > 12.367, *p*s < 0.001) and weak to moderate evidence against a main effect of group (BF_incl_ < 0.729, *F*s < 1.953, *p*s > 0.167). The only exception was strong evidence for a main effect of group [BF_incl_ = 14.919, *F*(1, 68) = 9.982, *p* = 0.002, *η*_p_^2^ = 0.128] on error rate on the different trials, with lower error rate in the mTBI group compared to the control group. In other words, the mTBI group did better on this aspect of the task compared to the control group. All analyses revealed weak to strong evidence against a degree of rotation × group interaction (BF_incl_ < 0.343, *F*s < 1.951, *p*s > 0.123).

Finally, our findings suggest that past mTBI does not negatively impact visual working memory performance (see Tables 3 and 4). A 2 (group) × 3 (set size) Bayesian repeated measures ANOVA on working memory capacity (K) showed very strong evidence for a main effect of set size, reflecting larger working memory load as set size increased. There was also moderate evidence for a main effect of group; however, it was the mTBI group that performed better than controls. Lastly, there was moderate evidence for a set size × group interaction. Follow-up independent samples t-tests revealed moderate evidence for a between-groups difference in performance at all three set sizes [set size 1: BF_10_ = 5.761, *t*(76) = − 2.751, *p* = 0.007, *d* = − 0.640; set size 2: BF_10_ = 5.072, *t*(76) =  − 2.693, *p* = 0.009, *d* = − 0.627; set size 3: BF_10_ = 4.184, *t*(76) =  − 2.605, *p* = 0.011, *d* = − 0.606]. These findings indicate the mTBI group performed better than the control group across all set sizes on the change detection task.

### Individual differences analyses

In our preregistration, we stated that we would conduct individual differences analyses for the mTBI group. Given our final sample size for this group lacks sufficient power to appropriately perform such analyses, these findings are provided in the Supplementary Material S1.

### Control analyses

Factors such as depression, anxiety, and post-concussion symptoms can influence cognitive performance^40–42^. Therefore, we ran a series of linear regressions with these variables, along with age, gender, education, and self-reported stress included in the null model. In each regression, the predictor was group and the outcome variable was the key dependent measure for the relevant task. There was moderate evidence for a relationship between group and performance on the change detection task [BF_10_ = 5.773, *R*^2^ = 0.158, *F*(10, 67) = 1.259, *p* = 0.272], with the mTBI group associated with better task performance. Moreover, there was weak evidence for an association between group and RMET performance [BF_10_ = 2.070, *R*^2^ = 0.190, *F*(10, 68) = 1.599, *p* = 0.126]. Again, the mTBI group was associated with better task performance. For all other tasks there was weak evidence against an association between group and task performance (BF_10_ < 0.680, *R*^2^ < 0.166, *F*s < 1.350, *p*s > 0.223). These analyses mirror the key findings reported above.

## Discussion

At present, the evidence regarding cognitive function in the chronic stage of mTBI is largely inconsistent and few rigorous, preregistered studies have been conducted in the general population. The current study used a wide variety of cognitively demanding behavioural tasks to investigate whether individuals with a history of mTBI show cognitive performance differences compared to control participants. To follow up the findings of Arciniega et al.^34^, we replicated their change detection paradigm to assess visual working memory. Expanding on past research, we also included measures of verbal working memory, multitasking ability, cognitive flexibility, response inhibition, visuospatial ability, information processing speed, and Theory of Mind.

Overall, our findings suggest, compared to control participants, a history of mTBI is not necessarily associated with cognitive performance deficits across any of the domains investigated. There was moderate evidence against between-group differences in performance on measures assessing verbal working memory, multitasking, cognitive flexibility, visuospatial ability, response inhibition, and information processing speed. In fact, there was weak evidence that the mTBI group performed better than controls on a measure of Theory of Mind and moderate evidence that the mTBI group performed better on a measure of visual working memory. Importantly, self-reported post-concussion symptoms, depression, and anxiety did not influence these results. While we conducted preliminary individual differences analyses in this study, further studies with larger samples are necessary to properly evaluate whether injury-related factors influence individual differences in cognitive performance in the chronic stage of injury.

These findings align with numerous past studies demonstrating that, at a group level, mTBI is not associated with long-term cognitive impairment^13,14,28,29^. While recent research by Arciniega et al.^34^ revealed evidence for prolonged visual working memory deficits in undergraduate students with a history of mTBI, we failed to replicate these findings in a Bayesian, preregistered study with a comparable sample size. Even though many mTBI participants in the current study had sustained multiple prior concussions, we found moderate evidence for better visual working memory performance in this group compared to controls across all set sizes. This indicates the original finding of a persistent working memory deficit in this population may not be robust.

Overall, this study provides evidence that even multiple occurrences of mTBI are not linked to long-term cognitive deficits. Yet, the injury remains a risk factor in later life for neurodegenerative diseases such as dementia and Parkinson’s disease^43^. Given we observed moderate evidence against performance differences on most cognitive tasks and moderate evidence for enhanced performance in the mTBI group on the change detection task, it is possible reliance on cognitive reserve may play a role in these findings^22^. Factors such as educational attainment and intelligence are hypothesised to enhance cognitive reserve^44,45^. Therefore, our mTBI sample consisting predominantly of high-functioning undergraduate university students are likely to have high levels of cognitive reserve. Broglio et al.^46^ speculate that young adults that have sustained a mTBI may rely on cognitive reserve and recruit alternative cerebral pathways to compensate for any subtle brain changes that occur following the injury. These compensatory processes allow individuals to maintain a high level of cognitive functioning, as we observed across all tasks in the current study, especially the visual working memory task. Future neuroimaging and longitudinal studies are necessary to further evaluate the cognitive reserve hypothesis in young adults with self-reported mTBI and how the aging process might interact with cognitive outcome post-injury.

Though the current study was preregistered, utilised Bayesian statistical analyses, and used largely complex behavioural tasks to assess a broad range of cognitive domains, a few limitations must be noted. Firstly, the time since the most recent head injury occurred and the number of past injuries sustained varied quite widely across participants in the mTBI group. However, this is not unusual for mTBI samples in the literature when not investigating single-incident mTBI (e.g., Arciniega et al.^34^). Secondly, it’s possible some of the tasks, particularly the Digit Span test^19^, may not have been sufficiently cognitively demanding enough to capture subtle performance differences between the two groups. Performance differences may be observed if a more complex measure of verbal working memory is used. Nonetheless, other measures we included were certainly sensitive enough to find group differences, but in the opposite direction to what has been reported previously. Lastly, the mTBI participants were aware they were being recruited due to their history of concussion. However, ‘diagnosis threat’ appears to negatively influence self-report measures of cognitive function, rather than objective measures of cognitive performance^47^. Therefore, this recruitment strategy is unlikely to have substantially impacted the study. While the current findings indicate no deficits in cognitive performance at the group level among participants with a history of mTBI, the individual differences analyses suggest injury-related factors may impact cognitive performance in the chronic mTBI population. Future studies should recruit a larger sample of chronic mTBI participants to properly evaluate the influence of these factors on cognitive performance at the individual level.

## Methods

### Preregistration

The study design and analysis plan were preregistered prior to data collection, and the materials and data are available online (https://osf.io/4v59y/).

### Participants

#### Recruitment and screening

Eighty-two participants were recruited via online advertisement and recruitment flyers posted around The University of Queensland campus and wider Brisbane catchment. While the advertisements stated that participants with a history of concussion were being recruited, the study was described as exploring “individual differences in cognitive performance” to mitigate diagnosis threat as much as possible.

Originally, participants were screened for mTBI history after completing the study. However, initial data collection indicated that mTBI participants were difficult to recruit. A screening questionnaire was implemented after testing the first 12 participants to ensure that eligible individuals for both the mTBI and control groups were being recruited. This change was amended in the preregistration document.

Inclusion criteria for the mTBI group conformed to the *American Congress of Rehabilitation Medicine*^48^ definition of the injury. These criteria are: loss of consciousness not exceeding 30 min, and/or posttraumatic amnesia not exceeding 24 h, and/or feeling dazed, disorientated or confused at the time of injury. Participants were excluded if their mTBI was sustained within three months of participating in the study (i.e., those not in the chronic stage of injury) or if they were hospitalised for longer than 24 h following injury.

Exclusion criteria for all participants included: history of psychotic or neurological disorder, current mood disorder or substance/alcohol dependence, and current use of psychiatric medications. While our preregistration stated that participants with a history of mood disorder would be excluded, it is most common in the literature to only exclude participants with a current mood disorder. Five participants in this study reported a past mood disorder; however, all scored moderate or lower on a self-report measure assessing depression, anxiety, and stress and none were currently taking medication. Therefore, they were included in the final sample. All participants reported normal or corrected to normal vision and hearing.

Two participants were excluded for meeting exclusion criteria and one participant was excluded due to technical difficulties during testing. Therefore, 79 participants were included in the final analyses, with 30 participants in the mTBI group and 49 participants in the control group. All participants provided written informed consent and received course credit or AU$20/h for their participation. The University of Queensland Human Research Ethics Committee (approval no. 2020000791) approved the study, all participants gave informed, written consent, and the study was conducted in accordance with the Declaration of Helsinki.

#### Bayesian sampling plan

In line with the Bayesian sampling approach, the sample size for this study was not predetermined. We recruited participants until a Bayes Factor BF_10_ > 3 or BF_10_ < 0.333 was established for five of the nine key dependent cognitive measures, indicating moderate evidence for the alternative or null hypothesis, respectively. A maximum sample size of 80 participants per group was also specified as a stopping rule for data collection. With that number of subjects, unequivocal findings would still be important for the field.

### Apparatus

The tasks were programmed in PsychoPy^49^ and MATLAB^50^ via the PsychToolbox extension^51,52^. Stimuli were presented on a 24-inch Asus LED monitor (resolution: 1920 × 1080, refresh rate: 60 Hz) using an Apple Mac Mini computer, with a viewing distance of approximately 57 cm. Participants responded to the self-report measures via Qualtrics.

### Procedure

Each session was conducted by a researcher who was blind to participants’ mTBI history. Participants first completed a battery of nine cognitive tasks, with four task orders counterbalanced across participants. Following the tasks, they responded to three self-report measures in a fixed order. Participants were fully debriefed at the end of the two-hour session.

### Tasks

The computerised task battery assessed the following domains: working memory, cognitive flexibility, multitasking, visual spatial ability, inhibition, information processing speed, and social cognition. All tasks are briefly described here, and in greater detail in the Supplementary Material S1.

#### Change detection task

**Figure 2:**
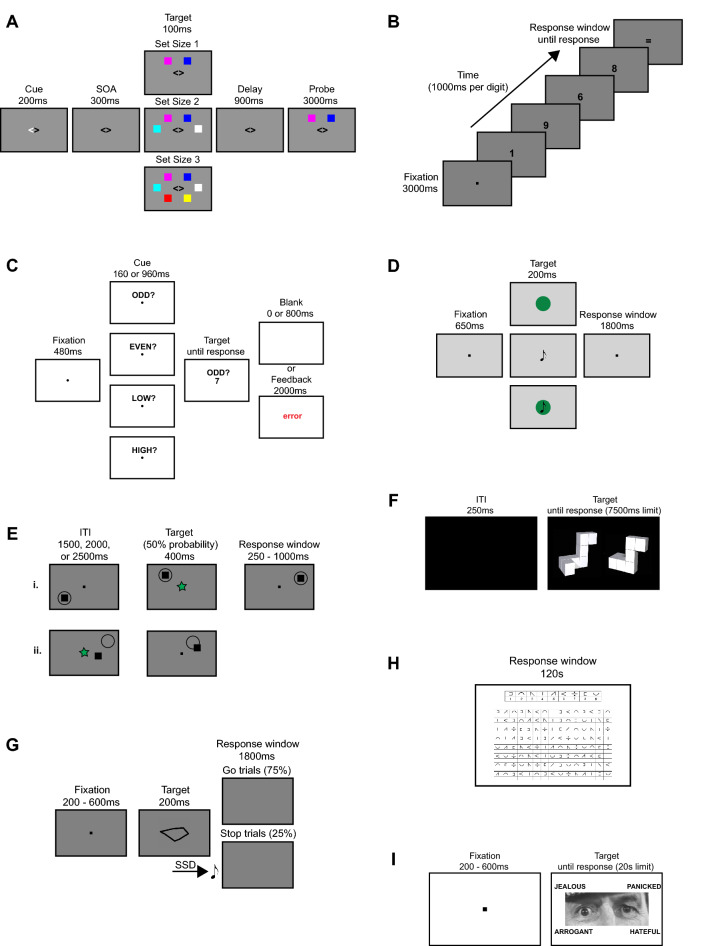
Schematic representations of the cognitive tasks. (**A**) Trial outline for the change detection task. (**B**) Trial outline for the Digit Span test. (**C**) Trial outline for the task switch paradigm. (**D**) Trial outline for the single vs dual task. (**E**) Trial outline for the dynamic dual task. (i) Trial outline for the shape discrimination task. (ii) Trial outline for the visuomotor tracking task. (**F**) Trial outline for the mental rotation task. (**G**) Trial outline for the stop-signal task. (**H**) Trial outline for the SDMT. (**I**) Trial outline for the RMET.

Visual working memory was assessed via a change detection task^34,53^. Participants were cued to attend to either the left or right hemifield, then one to three coloured squares were presented in each hemifield. Following a short retention interval, one probe item was presented in each hemifield (see Fig. 2A). Using a keypress response, participants determined whether the probe item in the cued hemifield matched the previously presented stimulus. Performance was assessed via working memory capacity [K = set size * (hit rate – false alarm rate)]^54,55^.

#### Digit span test

As a measure of verbal working memory, participants completed the Digit Span forward and backward tests^56^. Participants were presented with streams of single digits (see Fig. 2B) and recalled them in the order they were presented (forward condition) or in reverse order (backward condition). Performance was indexed as the highest number of digits accurately recalled in each condition, with the backward span score being the variable of interest.

#### Task switch paradigm

Cognitive flexibility was measured using a cued task switching paradigm (adapted from Experiment 6 in Monsell & Mizon^57^). Participants performed either a parity task (odd/even) or a magnitude task (smaller/larger than five) on the presented digit (one to nine, excluding five). A cue was displayed prior to the digit to indicate which task to perform (‘ODD?’ or ‘EVEN?’ for parity and ‘LOW?’ or ‘HIGH?’ for magnitude; see Fig. 2C). Responses were made via keypress. Switch cost (difference in response time between task-change and cue-change trials) was calculated to assess performance.

#### Single vs dual task

Multitasking was evaluated via a sensory motor decision-making paradigm^58^. This task involved auditory and visual decision-making tasks, presented either individually or concurrently (see Fig. 2D). Participants discriminated between the auditory and/or visual stimuli via keypress. A reaction time cost between single and dual task trials was calculated to assess performance.

#### Dynamic dual task

As a measure of continuous multitasking ability, participants completed a dual task that combines an ongoing visuomotor tracking task and a visual shape discrimination task^59^. The visuomotor tracking task involved using the mouse to continuously track a moving disk, while the shape discrimination task involved responding to the target shape stimulus via keypress (see Fig. 2E). Participants performed the tasks individually and concurrently. Performance was indexed via a combined multitasking accuracy cost between single and dual task trials for the two tasks.

#### Mental rotation task

Visual spatial ability was measured via a mental rotation task^60^. Participants determined whether pairs of three-dimensional objects presented at different angles of rotation were identical to one another or whether they were pseudo-mirror reflections (see Fig. 2F). Responses were made via keypress. Performance was assessed via reaction time at each angle of rotation.

#### Stop-signal task

As a measure of response inhibition, participants completed the stop-signal task used by Bender et al.^61^. Participants were instructed to discriminate between two abstract shapes via keypress on ‘go’ trials and withhold their response to the shapes on ‘stop-signal’ trials (see Fig. 2G). An auditory tone was presented after the shape stimulus to indicate the stop-signal trials. Stop-signal reaction time (see Supplementary Material S1) was used to assess performance.

#### Symbol digit modalities test

Information processing speed was assessed via a computerised Symbol Digit Modalities Test (SDMT)^62^. Participants were shown nine symbol-digit pairings and a list of 120 symbols (see Fig. 2H). They were given 120 s to enter the corresponding digit under each symbol using a keypress response. The total number of correct responses was recorded.

#### Reading the mind in the eyes test

Participants completed the Reading the Mind in the Eyes Test (RMET) as a measure of Theory of Mind^63^. Theory of Mind is a social-cognitive mechanism that refers to the ability to attribute mental states (e.g., beliefs, emotions, etc.) to ourselves and others. Participants were presented with an image of a person’s eyes and used a keypress response to select which of four words best described the person’s mental state (see Fig. 2I). Task accuracy was used to assess performance.

### Self-report measures

#### Health history questionnaire

The health history questionnaire collected information regarding participant demographics, mTBI history, and study exclusion criteria (e.g., medication use). Questions concerning mTBI history were adapted from previous researchers who investigated self-reported concussions in the general population^64,65^. Participants were presented with an initial question regarding their concussion history: ‘A concussion is defined as a blow to the head that forces one to stop whatever one is doing because of unconsciousness, dizziness, pain or disorientation. Based on this definition, have you ever had a concussion?’. If participants responded with yes, they were then asked to provide the following details regarding their injury (or injuries): (1) the number of concussions they had sustained; (2) how the injury occurred; (3) when (month and year) the injury occurred; (4) whether any loss of consciousness occurred and if yes, the estimated length; (5) whether any memory loss occurred and if yes, the estimated length; (6) whether any confusion or disorientation occurred and if yes, the estimated length; (7) whether medical attention was received and if yes, whether the injury was diagnosed as a concussion and how long they were hospitalised (if applicable). In line with the *American Congress of Rehabilitation Medicine*^48^ mTBI criteria, participants were not included in the mTBI group if they responded with yes to the initial concussion question but did not report any loss of consciousness, memory loss, or confusion/disorientation after the injury.

#### Rivermead post-concussion symptoms questionnaire (RPQ)

The RPQ^66^ measures the frequency and severity of 16 commonly experienced physical, emotional, and cognitive post-concussion symptoms. To allow the measure to be completed by both participants with a history of mTBI and controls, the questionnaire instructions were modified slightly. Participants were asked to indicate the degree to which they had experienced each symptom over the past 24 h, without connecting it to a previous head injury. Each item was rated on a five-point scale: 0 (not experienced at all), 1 (no more of a problem), 2 (a mild problem), 3 (a moderate problem), or 4 (a severe problem). Given the modified instructions, scores of 0 and 1 were both scored as 0 (not present). Adding all the item scores yields a total symptom score. The RPQ is a valid measure of post-concussion symptoms and has high reliability^66,67^.

#### Depression anxiety stress scales (DASS-21)

The DASS-21^68^ contains three seven-item self-report scales assessing depression, anxiety, and stress over the previous week. Each item was rated on a four-point severity scale ranging from 0 (never) to 3 (almost always). The DASS-21 is a valid and reliable measure of these emotional states^69^ and has demonstrated validity as a screening tool for depression and anxiety following TBI^70^.

### Data analyses

All data exclusions and analyses were preregistered. Outlier screening was performed for each participant, in each task. For the single vs dual, mental rotation, and stop-signal tasks, trials with a response time (RT) greater than three standard deviations from the mean or less than 200 ms were removed. For the task switch paradigm, trial exclusions included the initial practice trial in each block, trials following incorrect trials, and trials with RTs greater than 3000 ms or less than 200 ms. For the dynamic dual task, trials with accuracy or RT greater than three standard deviations from the mean were removed. Participants with poor performance on particular tasks were excluded from the analysis of that task. Specifically, those with less than 60% accuracy on dual task trials in the single vs dual task, less than 70% accuracy on the mental rotation task, less than 80% accuracy on the task switch paradigm, and accuracy greater than three standard deviations below the mean on the RMET or change detection task. Participants who were excluded for poor performance in one task were not necessarily excluded from other tasks, therefore sample size varied across tasks.

Trimmed data were analysed in JASP^71^ and R^72^ using Bayesian and frequentist statistical approaches. Interpretations were based on the Bayesian analyses, as these tests quantify the relative strength of evidence in favour of both the null and alternative hypotheses^23^. Here, the inverse Bayes Factor (BF_10_) is reported for simpler analyses and the inclusion Bayes Factor (BF_incl_) is reported for interaction effects. The BF_incl_ value indicates the relative evidence for the inclusion of each main effect and interaction in the model across matched models, providing a simpler interpretation of interaction effects. In line with standard interpretation of Bayes Factors^73^, values greater than 3 were considered moderate evidence for the alternative hypothesis, while values less than 0.333 indicate moderate evidence for the null hypothesis. Values between 1 and 3 or 0.333 and 1 were considered weak evidence for the alternative or null hypothesis, respectively. All analyses used default zero-centred Cauchy prior distributions.

## Supplementary Information

Supplementary Information.

## Data Availability

All data files are available at: https://osf.io/4v59y/.

## Code availability

Analysis code is provided at: https://osf.io/4v59y/.
