# Supplementary material for: The influence of self-reported history of mild traumatic brain injury on cognitive performance
Source: Sci Rep. 2022 Oct 11;12:16999. doi: 10.1038/s41598-022-21067-w (PMC9554181; doi:10.1038/s41598-022-21067-w)
Supplement: Supplementary file 1 — Supplementary Information. [file 41598_2022_21067_MOESM1_ESM.pdf]

The influence of self-reported history of mild traumatic brain injury on cognitive performance

Amaya J. Fox, Hannah L. Filmer and Paul E. Dux

Supplementary Information

Supplementary Methods

Change detection task[1,2]. Participants were required to determine whether a probe item was the same as the previously presented visual stimulus. The stimuli were different coloured squares (0.037w x 0.059h normalised units), randomly selected from a list of seven colours (white: RGB 255 255 255, blue: RGB 0 0 255, cyan: RGB 0 255 255, red: RGB 255 0 0, magenta: RGB 255 0 255, yellow: RGB 255 255 0, green: RGB 0 255 0). Each trial started with a central fixation cross (300ms, 0.022w x 0.035h normalised units) on a grey background (RGB 128 128 128). A left or right white arrowhead was then presented to indicate which hemifield to attend to (200ms, 0.093 normalised units). Following a brief delay (300ms), one to three stimuli were presented on each side of the fixation cross (100ms). After a short retention interval (900ms), a single probe item was presented in each hemifield (3000ms).

Participants determined whether the previously presented stimulus and the probe item in the cued hemifield were the same colour or different colours using a keypress response (“o” for same, “n” for different). Stimulus and probe items matched on 50% of trials. After a practice block of 24 trials, there were three blocks of test trials. Testing consisted of 288 trials, with 96 trials per set size (one, two, and three). The key dependent variable was working memory capacity [K = set size * (hit rate – false alarm rate)][3,4].

Digit Span test[5]. Participants were required to recall lists of digits in their presented order. In each trial, participants were presented with a central fixation square (3000ms, 0.01w x 0.01h height units) on a grey background (RGB 128 128 128). A stream of single digits were then displayed one at a time in Arial font in the centre of the screen (1000ms per digit, 0.08 height units). Finally, an equal sign (=) was centrally displayed, signalling participants to enter the digits they recalled using the relevant number keys on the keyboard.

In the forward span condition participants recalled the digits in the order they were presented, while in the backward span condition they recalled them in reverse order. If a correct response was made, the proceeding trial contained an additional digit. If two consecutive incorrect responses were made, the proceeding trial contained one less digit. The first trial in each condition consisted of two target digits to recall, followed by 14 test

trials. Participants' scores reflected the highest number of digits accurately recalled in each respective condition. The key dependent variable was the backward span score.

Task switch paradigm[6]. Participants were required to perform one of two simple tasks, based on the presented cue. Stimuli were the digits one to nine, excluding five. Participants made either a parity (odd or even) or magnitude (smaller or larger than five) judgement of the target digit, depending on the verbal cue displayed. Two verbal cues were assigned to each task: 'ODD?' and 'EVEN?' for the parity task and 'LOW?', and 'HIGH?' for the magnitude task. Cues were never repeated from one trial to the next, to avoid a confound between task changes and cue changes. In each trial, a central fixation dot (480ms, 0.07 normalised units) was displayed on a white background prior to the verbal cue (160ms or 960ms, cue-stimulus interval alternated each block) and remained onscreen until the target was presented. Cue and target (both Arial font, 0.1 normalised units) were displayed until a response was made. Following a response, a blank white screen was shown (800ms) if the cue-stimulus interval was 160ms, ensuring the response-stimulus interval was always 1440ms. If participants made an incorrect response, the word 'error' was presented in red (2000ms, 0.1 normalised units). Participants responded by pressing one of two keys, 'c' for low and odd digits or 'm' for high and even digits.

Practice consisted of two 16 trial blocks of the parity task alone, alternating with two blocks of the magnitude task alone. Every combination of cue and target occurred randomly within each block. Participants then completed 33 trials of mixed task practice, which mirrored the test blocks for the task. All practice trials began with a 300ms blank interval, followed by a 300ms fixation dot, and a 500ms cue prior to target presentation. Following the practice blocks, participants completed eight blocks of 33 trials. The first trial of each block was a warm-up trial, resulting in a total of 256 test trials. Within the blocks, each combination of cue and target occurred, and both trial types (task change and cue change) were equally represented. The key dependent variable was switch cost, calculated as the difference in response time on task-change and cue-change trials.

Single vs Dual task[7]. Participants completed two decision-making tasks, alone and concurrently, in this multitasking paradigm. The task involved three different trial types: single visual task, single auditory task, and visual auditory dual task. Visual stimuli consisted of a red (RGB 237 32 36), dark blue (RGB 44 71 151) and dark green (RGB 10 130 65) coloured circle (0.125w x 0.125h height units). Auditory stimuli included three complex tones played through headphones [7]. Trials began with a central fixation cross (650ms, 0.01w x 0.01h height units) displayed on a grey background (RGB 128 128 128), followed by the

presentation of the stimulus (200ms). On single task trials, either a coloured circle or a complex tone was presented, and participants identified the stimulus as quickly and accurately as possible. For the dual task trials, a coloured circle and a tone were presented simultaneously, and participants responded to both stimuli as quickly and accurately as possible. The response window was 1800ms.

Participants responded via keypress, with each individual stimulus corresponding to a specific key [‘A’, ‘S’, ‘D’ for the left hand and ‘J’, ‘K’, ‘L’ for the right hand (index fingers on ‘D’ and ‘J’)]. The response mappings were random across participants, with some participants using ‘A’, ‘S’, ‘D’ for the visual task and others using these keys for the auditory task. Participants first completed three practice blocks: 1) 15 single visual trials, 2) 15 single auditory trials, and 3) 10 single visual trials, 10 single auditory trials, and 10 visual auditory dual task trials randomly intermixed (30 trials total). In effort to reduce the number of exclusions for poor task performance, participants were required to reach an accuracy threshold of 70% on the dual-task trials before they could begin the test trials. Following practice, participants completed eight blocks of 30 test trials (i.e., 80 trials per trial type). The key dependent variable was multitasking response time cost between single and dual task trials.

Dynamic dual task[8]. Participants completed an ongoing visuomotor tracking task and a visual shape discrimination task, alone and concurrently, in this continuous multitasking paradigm. The task consisted of three trial types: single visuomotor tracking task, single shape discrimination task, and dual task. In the visuomotor tracking task, participants held the computer mouse in their right hand and continuously tracked a moving black target disk (0.50°) with the black mouse cursor (0.10°). Their aim was to keep the cursor as close as possible to the centre of the disk. At the beginning of each trial, the target disk was positioned at the top centre of the screen and the cursor was placed within the centre of the disk. Accuracy on the tracking task reflected the percentage of time the cursor stayed within the edges of the disk. In the shape discrimination task, participants were required to press the spacebar as quickly as possible when the target shape stimulus was presented on the screen. Stimuli (0.40°) consisted of 12 coloured (red: RGB 255 0 0, green: RGB 0 255 0, blue: RGB 0 0 255, and yellow: RGB 255 255 0) shapes (square, hexagon, and star). In each trial, a new target shape was randomly selected from the set, with the other coloured shapes presented as distractors. Participants were shown the target shape for the proceeding trial before it began. Shape stimuli were randomly displayed in the centre of the

screen (400ms), with the target shape occurring at 50% probability. Between each shape, a central fixation square (0.04°) was displayed (1500ms, 2000ms, or 2500ms).

On single task trials, participants performed either the visuomotor tracking task or the shape discrimination task. To hold visual stimulation constant across trial types, the moving target disk and shape stimuli were presented on every trial. When completing the visuomotor tracking task, participants were instructed to ignore the shape stimuli. Likewise, when performing the discrimination task, they were instructed to ignore the moving disc. On dual task trials, participants completed the visuomotor tracking task and shape discrimination task simultaneously. Participants first practiced each single task condition, which involved an adaptive thresholding procedure to establish an individual difficulty level that resulted in approximately 80% accuracy on each task. Specifically, if accuracy was greater than 82.5% or less than 77.5% at the end of a trial, the difficulty of the respective task was modified (see Bender et al.[8] for further details). Participants first completed nine 60 second visuomotor tracking practice trials, with continuous feedback. The cursor turned red if it was outside the radius of the moving target disk. Next, participants completed nine 20 second shape discrimination practice trials, with feedback. The central fixation square turned red (50ms) if participants responded too slowly to the target shape or they incorrectly responded to a non-target shape. Feedback was only provided during the practice trials. Following practice, participants completed five one-minute trials of each of the three trial types (15 test trials total). The key dependent variable was a combined multitasking accuracy cost between single and dual task trials for the two tasks.

Mental rotation task[9]. Participants were required to mentally rotate a target object (left object) and decide whether it was the same as, or a pseudo-mirror reflection of, the baseline object (right object). The stimuli were pairs of three-dimensional objects adapted from the original mental rotation stimuli. For this task, twelve baseline objects were randomly chosen from an open-source stimulus set[10]. The 12 objects were presented in both the 'same' and 'different' versions and at four different angles of rotation (0°, 50°, 100°, 150°), for a total of 96 stimulus pairs. Trials began with a blank black screen (250ms), followed by the stimulus (0.895w x 0.743h normalised units). The stimulus remained onscreen until a response was made (7500ms limit). Participants responded via keypress ('b' for same, 'n' for different). First, participants were given 12 practice trials with feedback. They then completed two blocks of 48 test trials. The key dependent variable was reaction time at each angle of rotation.

Stop-signal task[11]. Participants needed to withhold their motor response to visual stimuli on particular trials. Stimuli consisted of two abstract 2D shapes (100 x 100mm). Trials began with a central fixation square (200-600ms, randomly determined) on a grey background (RGB 128 128 128), followed by the shape stimulus (200ms). 'Go' trials (75% of trials) involved discriminating between the two shapes within the response window (1800ms). Participants responded via keypress, with each shape mapped to a response key ('f' or 'j'). On 'stop-signal' trials (25% of trials) participants withheld their response. To signify the stop-signal trials, an auditory tone (200ms, 750-Hz sine-wave tone) was presented after the shape stimulus.

To maintain a stopping probability of 50%, the interval between the presentation of the shape stimulus and the stop-signal (i.e., the stop-signal delay, SSD) was continuously modified via an adaptive staircasing procedure. The initial SSD was 250ms and increased by 50ms if participants successfully withheld their response or decreased by 50ms if participants failed to do so. After a practice block consisting of 24 trials with feedback, participants completed four test blocks of 36 trials. The key dependent variable was the stop-signal reaction time (SSRT; participants' mean go RT minus mean SSD), with lower SSRTs indicating better inhibition.

Symbol Digit Modalities Test (SDMT)[12]. Participants were instructed to insert corresponding digits below a series of symbols. They were presented with a reference key showing nine symbol-digit pairs and the list of symbols. The reference key was a 9 x 2 grid (0.83w x 0.22h normalised units) presented in the upper half of the screen, displaying the nine symbols in the top row and the nine corresponding digits (1 – 9) in the bottom row. In the lower half of the screen, a 15 x 16 grid (1.25w x 1.42h normalised units) was presented. This larger grid displayed the test symbols on the odd rows (i.e., rows 1, 3, 5 etc.) and empty boxes for the participant's responses on the even rows (i.e., rows 2, 4, 6, 8 etc.). Therefore, the test involved a total of 120 symbols. The symbols, digits and grid outlines were black, and the background was white. Using the relevant number keys on the keyboard, participants needed to enter the associated digit under each symbol as quickly and as accurately as possible. The task began with an untimed practice containing a list of six symbols. Participants were then given 120 seconds to respond to as many of the test symbols as possible. The key dependent variable was the total number of correct responses.

Reading the Mind in the Eyes Test (RMET)[13]. Participants must determine a person's mental state based on an image of their eyes. Each trial began with a brief fixation square

(200-600ms, randomly determined) on a white background, followed by one of 36 eye images from the original version of the task (until response, 20s limit; images ~150 x 160mm). The images were surrounded by four words describing mental states (e.g., ‘anxious’; Arial, size 20 font) and each word position had a corresponding response key (‘1’, ‘2’, ‘3’, and ‘4’). Participants selected the word that best described the thoughts or feelings of the person in the image. Following a single practice trial, participants completed the 36 test trials. The key dependent variable was accuracy, defined as the percentage of correct responses out of all responses.

Supplementary Results

Individual differences analyses. Though the mTBI sample in this study is likely insufficient to perform individual differences analyses, we conducted these analyses as per our preregistration. To assess the predictive value of injury-related variables for individual differences in cognitive performance within the mTBI group, a series of linear regressions were run. The injury-related predictors included RPQ scores, time since most recent injury, number of past head injuries, cause of injury (i.e., sports-related, accidental fall, car accident, interpersonal violence, or head striking object), and whether medical attention was received. The outcome variable was the key dependent measure for each cognitive task. We assessed the collinearity of the predictor variables via the variance inflation factor (VIF). All VIF values were below 5; therefore, collinearity between variables was not an issue in these analyses[14]. Three sets of regressions were run for each outcome variable: 1) for the main analysis, the injury-related factors were included as predictors, with the participant characteristics of age, gender, education level (high school, Bachelor's degree, Master's degree, Diploma), depression, anxiety, and stress included in the null model; 2) the same analyses were run with no participant characteristics included in the null model, to determine whether these variables influence the findings; and 3) only age, gender, education, depression, anxiety, and stress were entered as predictors, to assess whether these factors impacted cognitive performance. To determine whether any factors of interest in each analysis uniquely contributed to cognitive performance, each factor was, in turn, entered as a predictor with all other variables included in the null model.

Change detection task. No models in the analyses showed more than weak evidence for a relationship with task performance [$BF_{10} < 2.320$, $R^2 < .229$, *F*s < 4.018, *p*s > .030]. Thus, no injury-related or participant characteristics appear to predict performance on the change detection task.

Digit Span test. In the main analysis, the model with the highest evidence included two factors: accidental fall injuries and medical attention [$BF_{10} = 3.193$, $R^2 = .708$, $F(10, 17) = 4.128$, $p = .005$]. Specifically, accidental falls compared to sports-related injuries and not receiving medical attention were associated with better task performance. No injury-related factors provided more than weak evidence for an independent contribution to Digit Span performance ($BF_{10} < 1.878$, *p*s > .091). When no factors were included in the null model, the model including accidental fall injuries and medical attention again gave the highest level of evidence [$BF_{10} = 10.654$, $R^2 = .351$, $F(2, 25) = 6.756$, $p = .005$]. There was strong evidence that medical attention made a unique contribution to task performance ($BF_{10} = 22.311$, $p =$

.002). No other injury-related variables showed more than weak evidence for an independent relationship with performance ($BF_{10} < 1.645$, *p*s > .094). When only participant characteristics were included as predictors, the model with the highest evidence included one factor: a Master's degree level of education [$BF_{10} = 64.287$, $R^2 = .365$, $F(1, 28) = 16.079$, $p < .001$]. Having a Master's degree compared to a high school level of education was associated with better performance on the Digit Span test. While there was moderate evidence for a Master's degree level of education making a unique contribution to cognitive performance ($BF_{10} = 9.414$, $p = .006$), no other participant characteristics were independently related to performance ($BF_{10} < 0.815$, *p*s > .314).

Single vs dual task. No models in the analyses showed more than weak evidence for a relationship with task performance [$BF_{10} < 2.704$, $R^2 < .442$, *F*s < 1.082, *p*s > .434]. Thus, no injury-related or participant characteristics appear to predict performance on the single vs dual task.

Dynamic dual task. The model with the highest evidence in the main analysis included the following factors: RPQ scores, number of past head injuries, and injuries involving the head striking an object [$BF_{10} = 1359.611$, $R^2 = .790$, $F(11, 16) = 5.467$, $p = .001$]. Higher RPQ scores and more past head injuries were associated with worse performance on the dynamic dual task, while the head striking an object compared to sports-related injuries was associated with better performance. There was moderate and strong evidence that RPQ scores ($BF_{10} = 4.001$, $p = .029$) and number of past head injuries ($BF_{10} = 23.869$, $p = .003$), respectively, made a unique contribution to dynamic dual task performance. No other injury-related variables revealed more than weak evidence for an independent relationship with performance ($BF_{10} < 1.428$, *p*s > .125). With no factors included in the null model, the model with the highest evidence included two factors: time since most recent injury and number of past head injuries [$BF_{10} = 69.341$, $R^2 = .458$, $F(2,27) = 11.142$, $p < .001$]. While there was strong evidence for number of past concussions uniquely contributing to dynamic dual task performance ($BF_{10} = 11.094$, $p = .005$), no other factors showed more than weak evidence for an independent relationship with performance ($BF_{10} < 1.360$, *p*s > .128). When only participant characteristics were entered as predictors, no models showed evidence for a relationship with dynamic dual task performance ($BF_{10} < 0.521$, $R^2 < .038$, *F*s < 1.098, *p*s > .304) and no factors independently contributed to performance ($BF_{10} < 0.895$, *p*s > .374).

Mental rotation task. In the main analysis, the model with the highest evidence included three factors: time since most recent injury, number of past head injuries, and injuries involving the head striking an object [$BF_{10} = 48.720$, $R^2 = .854$, $F(11, 13) = 6.926$, $p < .001$].

Longer time since injury and a greater number of past head injuries were associated with longer response time on the mental rotation task, while the head hitting an object compared to sports-related injuries was related to faster response time. No injury-related predictors showed more than weak evidence for an independent relationship with task performance (BF_{10} < 2.119, *p*s > .068). When no factors were entered into the null model, no models showed more than weak evidence for a relationship with mental rotation performance (BF_{10} < 1.408, R^2 < .224, *F*s < 3.123, *p*s > .063) and no injury-related predictors showed more than weak evidence for an independent relationship with task performance (BF_{10} < 1.306, *p*s > .163). In contrast, when only participant characteristics were entered as predictors, the model including gender, anxiety, and stress showed the highest evidence [BF_{10} = 16.207, R^2 = .463, $F(3, 22) = 6.334$, $p = .003$]. Male participants and participants with higher anxiety responded faster on the mental rotation task, while higher stress was associated with longer response time. There was strong evidence that gender uniquely contributed to mental rotation performance (BF_{10} = 15.015, $p = .003$), but no other participant characteristics showed more than weak evidence for an independent relationship with performance (BF_{10} < 1.945, *p*s > .068).

RMET. The model with the highest evidence in the main analysis included the factors time since most recent injury, accidental fall injuries, interpersonal violence injuries, and medical attention [BF_{10} = 13.204, R^2 = .650, $F(12, 15) = 3.325$, $p = .062$]. Specifically, longer time since injury, receiving medical attention, and interpersonal violence compared to sports-related injuries were associated with worse RMET performance. In contrast, accidental falls compared to sports-related injuries was related to better performance. While there was moderate evidence that accidental fall injuries uniquely contributed to task performance (BF_{10} = 6.358, $p = .019$), no other injury-related variables showed more than weak evidence for an independent relationship with performance (BF_{10} < 1.548, *p*s > .143). With no factors included in the null model, the model including the factors number of past head injuries and accidental fall injuries showed the highest evidence [BF_{10} = 7.227, R^2 = .326, $F(2, 25) = 6.033$, $p = .007$]. Again, there was moderate evidence that accidental fall injuries uniquely contributed to RMET performance (BF_{10} = 3.330, $p = .030$), but no other injury-related factors were independently associated with performance (BF_{10} < 1.046, *p*s > .203). When only participant characteristics were entered as predictors, no models showed evidence for a relationship with RMET performance (BF_{10} < 0.965, R^2 < .090, *F*s < 2.782, *p*s > .106) and no factors showed more than weak evidence for independently contributing to performance (BF_{10} < 2.199, *p*s > .064).

SDMT. In the main analysis, the model with the highest evidence included four factors: accidental fall injuries, injuries involving the head striking an object, car accident injuries, and medical attention [BF_{10} = 5.466, R^2 = .563, $F(12, 15) = 1.610$, p = .190]. Compared to sports-related injuries, injuries involving accidental falls and the head striking an object were associated with better SDMT performance. Conversely, car accident compared to sports-related injuries and receiving medical attention were associated with worse SDMT performance. There was moderate evidence that accidental fall injuries (BF_{10} = 3.373, p = .052) and medical attention (BF_{10} = 8.328, p = .016) uniquely contributed to task performance. No other injury-related variables were independently related to performance (BF_{10} < 1.569, p > .158). When no factors were entered into the null model, the model including accidental fall injuries and medical attention revealed the highest evidence [BF_{10} = 5.425, R^2 = .306, $F(2, 25) = 5.511$, p = .010]. With only participant characteristics were included as predictors, no models showed evidence for a relationship with SDMT performance (BF_{10} < 0.992, R^2 < .164, Fs < 2.655, ps > .089) and no factors showed more than weak evidence for independently contributing to performance (BF_{10} < 1.283, ps > .172).

Stop signal task. The model with the highest evidence in the main analysis included the following factors: RPQ scores, time since most recent injury, number of past head injuries, accidental fall injuries, and injuries involving the head striking an object [BF_{10} = 37.893, R^2 = .662, $F(13, 14) = 2.113$, p = .089]. Greater RPQ scores, time since most recent injury, and number of past head injuries, as well as injuries from accidental falls and the head striking an object compared to sports-related injuries were associated with better stop-signal task performance (i.e., faster response time). However, no injury-related factors showed more than weak evidence for an independent relationship with task performance (BF_{10} < 2.800, ps > .059). When no factors were entered into the null model, the model showing the highest evidence included only two factors: RPQ scores and time since most recent injury [BF_{10} = 3.273, R^2 = .270, $F(2, 27) = 3.203$, p = .056]. Again, no injury-related factors showed more than weak evidence for a unique association with task performance (BF_{10} < 1.436, ps > .122). When participant characteristics were entered as the only predictors, no models showed evidence for a relationship with stop signal performance (BF_{10} < 0.467, R^2 < .028, Fs < 0.807, ps > .377). No factors showed more than weak evidence for independently contributing to performance (BF_{10} < 1.019, ps > .286).

Task switch paradigm. In the main analysis, the model with the highest evidence included two factors: accidental fall injuries and car accident injuries [BF_{10} = 3.141, R^2 = .521, $F(10, 17) = 1.852$, p = .127]. Specifically, accidental fall and car accident compared to sports-related injuries were associated with better task switch performance (i.e., reduced switch

cost). No injury-related factors showed more than weak evidence for an independent relationship with task performance ($BF_{10} < 1.347$, *p*s > .200). With no factors in the null model, no models showed evidence for a relationship with stop signal performance ($BF_{10} < 0.986$, $R^2 < .098$, *F*s < 2.811, *p*s > .106) and no injury-related predictors revealed more than weak evidence for a unique association with performance ($BF_{10} < 1.246$, *p*s > .172). With only participant characteristics included as predictors, the model with the highest evidence included two factors: gender and depression [$BF_{10} = 5.870$, $R^2 = .293$, $F(2, 27) = 5.595$, $p = .009$]. Male participants and participants with higher depression performed worse on the task switch paradigm. While there was moderate evidence that gender uniquely contributed to task switch performance ($BF_{10} = 5.661$, $p = .013$), no other factors were independently related to performance ($BF_{10} < 0.982$, $p > .248$).

While the current sample size of mTBI individuals is insufficient for individual differences analyses, the findings from these analyses suggest injury-related factors such as cause of injury, number of past head injuries, and whether medical attention was received may impact cognitive performance.

Supplementary References

1. Vogel, E. K. & Machizawa, M. G. Neural activity predicts individual differences in visual working memory capacity. *Nature* **428**, 748-751 (2004).
2. Arciniega, H. *et al.* Visual working memory deficits in undergraduates with a history of mild traumatic brain injury. *Attention, Perception, & Psychophysics* **81**, 2597-2603 (2019).
3. Cowan, N. The magical number 4 in short-term memory: A reconsideration of mental storage capacity. *Behavioral and brain sciences* **24**, 87-114 (2001).
4. Pashler, H. Familiarity and visual change detection. *Perception & psychophysics* **44**, 369-378 (1988).
5. Woods, D. L. *et al.* Improving digit span assessment of short-term verbal memory. *Journal of clinical and experimental neuropsychology* **33**, 101-111 (2011).
6. Monsell, S. & Mizon, G. A. Can the task-cuing paradigm measure an endogenous task-set reconfiguration process? *Journal of Experimental Psychology: Human Perception and Performance* **32**, 493-516 (2006).
7. Filmer, H. L., Lyons, M., Mattingley, J. B. & Dux, P. E. Anodal tDCS applied during multitasking training leads to transferable performance gains. *Scientific Reports* **7**, 12988 (2017).
8. Bender, A. D., Filmer, H. L., Naughtin, C. K. & Dux, P. E. Dynamic, continuous multitasking training leads to task-specific improvements but does not transfer across action selection tasks. *npj Science of Learning* **2**, 14 (2017).
9. Shepard, R. N. & Metzler, J. Mental rotation of three-dimensional objects. *Science* **171**, 701-703 (1971).
10. Ganis, G. & Kievit, R. A new set of three-dimensional shapes for investigating mental rotation processes: Validation data and stimulus set. *Journal of Open Psychology Data 3:e3* (2015).
11. Bender, A. D., Filmer, H. L., Garner, K., Naughtin, C. K. & Dux, P. E. On the relationship between response selection and response inhibition: An individual differences approach. *Attention, Perception, & Psychophysics* **78**, 2420-2432 (2016).
12. Hinton-Bayre, A. & Geffen, G. Comparability, reliability, and practice effects on alternate forms of the Digit Symbol Substitution and Symbol Digit Modalities tests. *Psychological assessment* **17**, 237 (2005).
13. Baron-Cohen, S., Wheelwright, S., Hill, J., Raste, Y. & Plumb, I. The "Reading the Mind in the Eyes" Test revised version: a study with normal adults, and adults with Asperger

syndrome or high-functioning autism. *The Journal of Child Psychology and Psychiatry and Allied Disciplines* **42**, 241-251 (2001).

14. Hair, J. F., Ringle, C. M. & Sarstedt, M. PLS-SEM: Indeed a silver bullet. *Journal of Marketing theory and Practice* **19**, 139-152 (2011).
